# The subdural membrane: unraveling the pathophysiology and treatment of chronic subdural hematoma

**DOI:** 10.3389/fsurg.2025.1694048

**Published:** 2025-12-09

**Authors:** Naveen Arunachalam Sakthiyendran, Jimena Gonzalez-Salido, Teresia Perkins, Alejandro Enriquez-Marulanda, Hormuzdiyar Dasenbrock, James Holsapple

**Affiliations:** 1 Department of Neurosurgery, Boston Medical Center, Boston University Chobanian and Avedisian School of Medicine, Boston, MA, United States; 2 Division of Neurosurgery, Beth Israel Deaconess Medical Center, Harvard Medical School, Boston, MA, United States

**Keywords:** subdural hematoma, dexamethasone, atorvastatin, burr holes, pathophysiology

## Abstract

Chronic subdural hematoma (cSDH) is one of the most common neurosurgical conditions, with incidence expected to rise sharply as populations age. Once regarded as a passive sequela of venous rupture, cSDH is now understood as an active, membrane-driven disorder characterized by persistent inflammation, fragile neovascularization, and local fibrinolysis that collectively fuel hematoma expansion and recurrence. This evolving biological model explains the limitations of traditional surgical approaches, which relieve mass effect but do not address the underlying pathophysiology. Burr-hole drainage with subdural drainage remains the gold standard, though recurrence rates approach 10%–20% and perioperative risks are magnified in frail patients. Minimally invasive strategies, such as twist-drill craniostomy and the subdural evacuating port system, expand options for high-risk populations, while mini-craniotomy may benefit complex or organized collections. Emerging adjuvant therapies, including middle meningeal artery embolization, statins, tranexamic acid, and corticosteroids, target the inflammatory and angiogenic milieu; however, results remain heterogeneous, and safety concerns persist. Novel directions such as biomarker-guided patient stratification, anti-VEGF therapies, platelet-rich plasma injection, and endoscopic membranectomy reflect a paradigm shift toward disease-modifying interventions. Collectively, these advances highlight a growing opportunity to personalize treatment, reduce recurrence, and improve long-term outcomes in cSDH.

## 1 Introduction

Chronic subdural hematoma (cSDH) is among the most common neurosurgical conditions, particularly affecting elderly patients. Its incidence is estimated to range from 1.7 to 58 cases per 100,000 persons annually, and is projected to rise substantially in the coming decades with the aging global population (1, 2). Remote head trauma is thought to be the most common cause, with recognized risk factors including advanced age, male sex, alcoholism, and the use of anticoagulant or antiplatelet medications (3).

Management can be either conservative or surgical, depending on the patient's overall condition, clinical presentation, and hematoma volume (>10 mm). Although surgical evacuation is often considered the first-line treatment for symptomatic patients, it carries the risk of neurological complications and a high recurrence rate (3, 4). Consequently, several pharmacological agents, including dexamethasone (DXA), atorvastatin, and tranexamic acid (TXA), have been investigated as adjuvant or alternative therapies; however, their efficacy remains the subject of ongoing debate (2).

Here, we review the pathophysiology and integrated management of cSDH, emphasizing current treatment paradigms, emerging therapeutic strategies, and areas of ongoing investigation.

## 2 Subdural membranes formation

### 2.1 The subdural space

Under normal conditions, the dura mater, the intervening dural border cell (DBC) layer, and the arachnoid barrier cell layer are well apposed to each other, to the point that the dura and arachnoid layers are almost in contact, and no true “subdural space” exists. The DBC layer is composed of flattened fibroblast-like cells embedded in sparse extracellular matrix, with weak intercellular junctions that make it structurally loose (5–9). Cleavage of the DBC layer by any accumulation of fluid that follows the path of least resistance can expand this potential space (9, 10). Once the fluid reaches a certain volume, it becomes a subdural collection in the subdural space.

### 2.2 The origin of a subdural hematoma

Disruption may occur from traumatic mechanisms such as minor head injury, falls, or whiplash, particularly in elderly patients with cerebral atrophy. Non-traumatic mechanisms include anticoagulation or antiplatelet therapy, and spontaneous or iatrogenic intracranial hypotension (11). Age-related tissue fragility and comorbidities further predispose the DBC layer to cleavage (12, 13). As discussed previously, when the DBC layer is disrupted, the resulting cleavage becomes a locus for fluid accumulation. This space can be seeded by blood and plasma from injured bridging veins, ruptured dural vascular plexus vessels, microvascular leakage, CSF, or a protein-rich hygroma (9, 11, 14). Any fluid collection in this space serves as a scaffold for cellular infiltration and the organization of membranes.

### 2.3 Mechanisms of SDH persistence and evolution

While cSDH was long viewed as a passive accumulation of blood from bridging-vein rupture, recent evidence has demonstrated that it is driven by persistent inflammation and pathological neoangiogenesis with leaky capillaries within the subdural membranes (9, 15, 16).

Damage-associated molecular patterns (DAMPs) released from injured tissue initiate a potent inflammatory cascade within the subdural space, triggering the recruitment of neutrophils, monocytes/macrophages, and lymphocytes (17, 18). Macrophages exhibit a mixed M1/M2 activation profile, contributing to both proinflammatory signaling and tissue remodeling. Elevated proinflammatory cytokines, including IL-6, IL-1β, TNF-α, and IL-8, promote endothelial permeability, leukocyte adhesion, and sustained cellular infiltration (9). Activated fibroblasts, stimulated by transforming growth factor-β (TGF-β) and other growth factors, deposit extracellular matrix (ECM) proteins, including collagen and fibronectin, progressively organizing the hematoma cavity into distinct outer and inner vascularized neomembranes (9).

The outer membrane is highly vascular and biologically active, containing proliferating fibroblasts, inflammatory cells (including neutrophils, lymphocytes, macrophages, and eosinophils), and numerous fragile capillaries (9). These neocapillaries are “leaky”; they exhibit thin or absent basement membranes and abundant gap junctions, resulting in high permeability and continuous protein-rich fluid flow into the subdural space, which fuels the expansion of the collection (9, 15). The inner membrane, adjacent to the arachnoid, acts as a semipermeable, mostly avascular collagenous barrier that impedes hematoma resorption (5) (Table 1).

**Table 1:** Key mediators and pathways in cSDH pathobiology and therapeutic implications.

Mediator/pathway [Reference(s)]	Role in CSDH	Direction vs. serum	Therapeutic implication
IL-6, IL-1β, TNF-α, IL-8 (9, 12, 18, 43, 46, 47)	Pro-inflammatory signaling, endothelial activation, leukocyte recruitment	Elevated in hematoma fluid	Rationale for anti-inflammatory strategies; avoid routine systemic steroids given harms
VEGF (9, 21, 22, 24, 32–34, 39, 70, 74)	Endothelial proliferation, junctional disruption → leakiness	Markedly elevated (often >20×)	Supports anti-angiogenic approaches; statin pleiotropy; investigational anti-VEGF
Angiopoietin-2 (↑)/Ang-1 (↓) (9, 21, 22)	Vessel destabilization and sprouting	Ang-2 predominance	Target Tie2 axis; complements VEGF pathway targeting
MMP-1/-2/-9 (9, 23, 24)	ECM and junction protein degradation; vessel leakiness	Elevated; correlate with VEGF	Consider MMP modulation; explains friable membrane
tPA/uPA, thrombomodulin (2, 3, 12, 25, 42, 46)	Hyperfibrinolysis, liquefaction, impaired stable clot	Elevated	Biological rationale for TXA
Heme/iron → HO-1 (9, 10)	Oxidative injury sustaining inflammation	Increased	Antioxidant/iron-handling targets (conceptual)

Biochemically, hematoma fluid exhibits a coagulation–fibrinolysis imbalance, with elevated tissue plasminogen activator (tPA) and urokinase plasminogen activator (uPA) levels, which prevent stable clot formation (19). Oxidative stress from red blood cell breakdown releases heme and iron, inducing heme oxygenase-1 and perpetuating membrane inflammation (20).

Mechanically, in brains with age-related atrophy, the enlarged subdural space facilitates reaccumulation without a marked elevation in intracranial pressure (10). This combination of persistent inflammation, ongoing angiogenesis, impaired coagulation, oxidative injury, and mechanical predisposition explains the persistence, evolution, and high recurrence rate of cSDH.

### 2.4 Angiogenic factors

The cSDH fluid and membranes harbor elevated levels of pro-angiogenic mediators that drive neovascular proliferation and leakage. Vascular endothelial growth factor (VEGF) is one of the key players in this angiogenic phase, as shown by hematoma fluid VEGF concentrations being dramatically elevated than those in serum (often >20-fold) (21).VEGF induces endothelial proliferation and junctional disruption, increasing microvascular permeability. Excess VEGF in cSDH correlates with radiological features of repeated bleeding (e.g., “mixed-density” or multilayered collections on CT/MRI), and outer-membrane VEGF expression has been linked to the risk of recurrence after surgical evacuation (21). Targeting VEGF signaling has therefore been proposed as a potential adjuvant to reduce the recurrence of disease.

Another angiogenic pathway is the angiopoietin/Tie2 system: the outer membrane overexpresses angiopoietin-2 (Ang-2) relative to angiopoietin-1 (Ang-1), tipping the balance toward vessel instability and sprouting of new, fragile capillaries. High Ang-2 further promotes permeability, compounding the VEGF effect (22).

Matrix metalloproteinases (MMPs) are also abundant in cSDH and contribute to membrane remodeling and vessel leakiness. Specifically, MMP-1, MMP-2, and MMP-9 have been identified in subdural fluid and outer membranes at much higher levels than in blood. Their proteolytic activity degrades extracellular matrix and endothelial junction proteins, facilitating angiogenesis but rendering the newly formed vessels structurally compromised (23). Moreover, MMP-2 and MMP-9 levels strongly correlate with VEGF in cSDH fluid, suggesting a synergistic angiogenic process that fuels hematoma growth (24).

**Figure 1:**
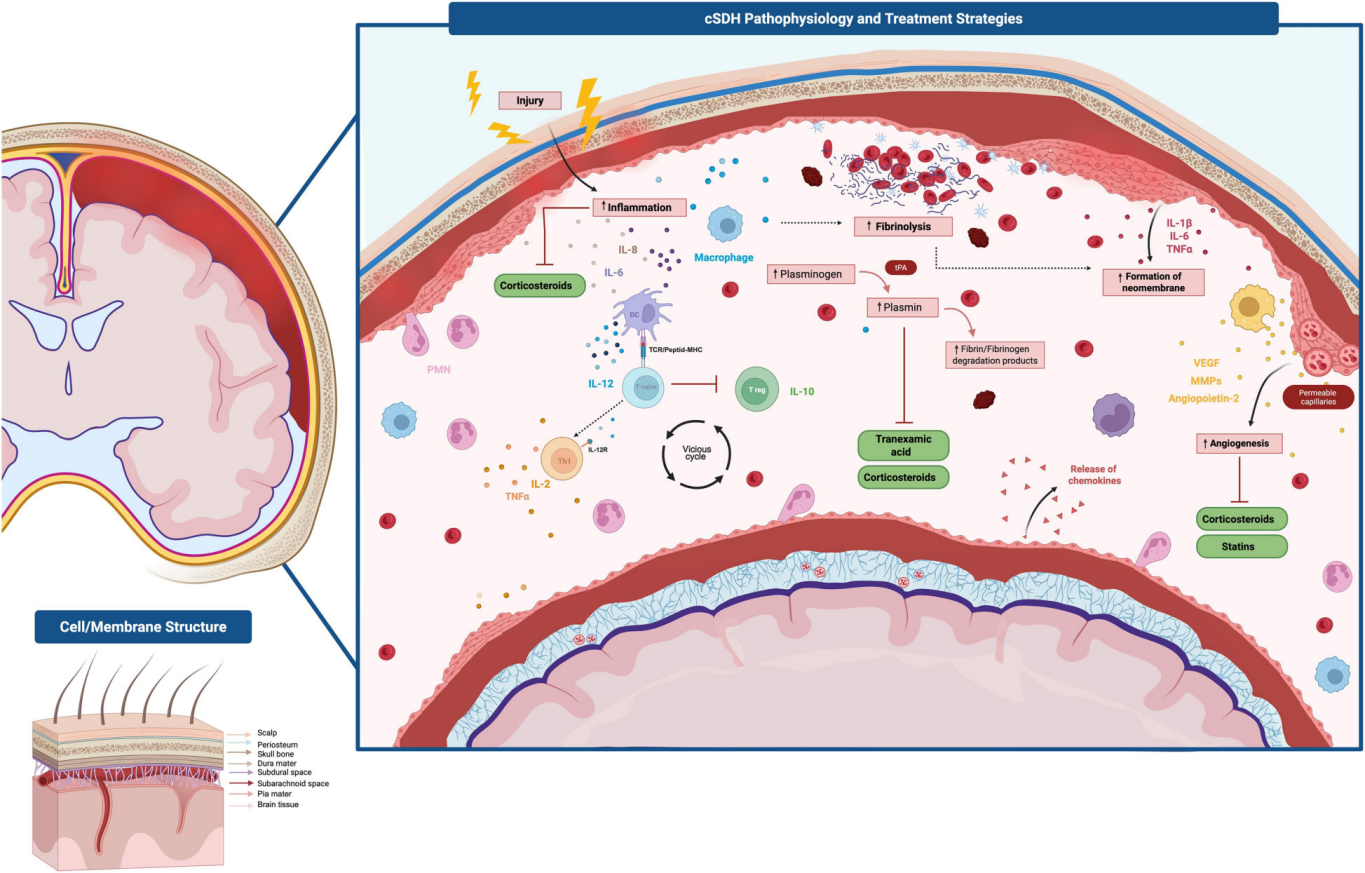
Key pathological mechanisms and emerging treatment strategies in chronic subdural hematoma (cSDH). Following disruption of the dural border cell layer from trauma or coagulopathy, a cascade of inflammation, fibrinolysis, and angiogenesis drives hematoma formation, persistence, and recurrence. Inflammatory mediators (IL-1β, IL-6, TNF-α) promote recruitment of neutrophils and macrophages, while increased fibrinolytic activity mediated by plasmin leads to liquefaction and expansion of the hematoma. The outer neomembrane becomes highly vascularized, characterized by fragile, leaky capillaries supported by VEGF, MMPs, and angiopoietin-2 signaling, perpetuating re-bleeding. This cycle of inflammation and angiogenesis fosters the chronic, relapsing nature of the disease. Therapeutic strategies target these mechanisms: corticosteroids modulate inflammation and angiogenesis, tranexamic acid suppresses fibrinolysis, and statins stabilize endothelial function and reduce neovascular permeability. Middle meningeal artery embolization interrupts vascular supply to the outer membrane, further disrupting the cycle of recurrence. *Gonzalez-Salido J. 2025* Created with Biorender.

High concentrations of interleukin-6 (IL-6) and IL-8, potent pro-angiogenic and neutrophil-recruiting cytokines, are also consistently found in cSDH fluid, far exceeding serum levels (9). Other mediators include tumor necrosis factor-α (TNF-α) and anti-inflammatory IL-10, reflecting a complex immune response balancing repair and inflammation (9). In addition, high levels of fibrinolytic factors, such as tissue plasminogen activator (tPA) and thrombomodulin, in outer membranes contribute to local coagulopathy and hematoma liquefaction (25). Together, inflammation-driven angiogenesis, proteolytic remodeling, and hyperfibrinolysis converge to create a fragile neomembrane ecosystem prone to expansion and recurrence. This pathophysiological process is detailed in Figure 1.

In addition to VEGF, Ang/Tie2, and MMPs, Autotaxin (ATX)–lysophosphatidic acid (LPA) signaling is newly implicated: a prospective clinical study demonstrated ∼3-fold higher ATX catalytic activity in hematoma fluid vs. paired serum, directly implicating the ATX–LPA axis in cSDH pathophysiology. LPA receptors (e.g., LPA1/3) promote endothelial permeability and fibroblast activation, offering a plausible mechanistic link between membrane inflammation and exudation (26). While clinical cSDH trials are lacking, LPA-directed strategies (e.g., ATX inhibitors, anti-LPA antibodies) have shown vascular-stabilizing effects in preclinical brain-injury models and represent a rational future avenue for study in cSDH (27). COX-2–prostaglandin E2 (PGE2) signaling may also intersect angiogenesis in cSDH. COX-2–positive cells have been identified in the outer membrane, PGE2 concentrations are significantly higher in hematoma fluid than serum, and PGE2 levels correlate with hematoma “age” (28).

### 2.5 Implications for the management of cSDH

Recognition of the biological activity of neomembranes underscores the rationale for treatments that target their vascular supply, inflammatory profile, or fibrinolytic activity. These include middle meningeal artery embolization, anti-inflammatory or anti-angiogenic pharmacotherapy, and agents that modulate fibrinolysis (14, 29–31).

## 3 Statin use for cSDH treatment

In the past decade, HMG-CoA reductase inhibitors (statins) have been investigated as a potential medical therapy for cSDH, based on their pleiotropic effects on endothelial integrity, inflammation, and angiogenesis. Preclinical studies have demonstrated that atorvastatin and simvastatin upregulate angiogenic growth factors, enhance pericyte coverage of neovessels, and reduce proinflammatory cytokines, thereby potentially promoting hematoma resorption (21, 32).

### 3.1 Pilot prospective study

Jiang et al. conducted an open-label, prospective cohort study in 2016 enrolling 23 patients with small-to-moderate cSDH who declined or were poor candidates for surgery. Patients received oral atorvastatin for 8 weeks. By 1 month, mean hematoma volume decreased from 49.3 ± 12.7 mL to 17.2 ± 8.5 mL, at 2 months, 59% (13/22) achieved ≥80% volume reduction, and by 3 months, 77% (17/22) had complete radiographic resolution (the remaining 5 patients demonstrated >70% reduction). No statin-related adverse events (e.g., myopathy, hepatic dysfunction) were observed during up to 36 months of follow-up, and functional scores (Markwalder grading scale) improved by ≥1 point in 91% of patients at 3 months (33) (Table 2).

**Table 2:** Pharmacologic therapies in cSDH—summary of evidence.

Therapy	Key trials/syntheses	Regimen (per study)	Primary effect	Recurrence/Surgery	Functional outcome	Safety signals
Atorvastatin (33–35)	ATOCH Phase II RCT, *n* = 200	20 mg daily × 8 wks	−12.5 mL greater volume reduction vs. placebo at 8 wks	Lower surgical rescue vs. placebo	Higher neurological improvement (45.9% vs. 28.6%)	Well tolerated; no ↑ serious AEs
Atorvastatin (33)	Open-label cohort, *n* = 23	8 wks	Marked volumetric reduction by 1–3 mo	77% complete radiographic resolution by 3 mo	MGS improved ≥1 point in 91%	No statin-related AEs
TXA (2, 3, 42)	Meta-analysis (2024)	Various; often oral TXA post-op	↓ Recurrence (OR: 0.35, *p* < 0.01)	↓ Hematoma volume at 3 mo	—	No ↑ AEs (OR: 1.84, *p* = 0.42)
TXA (1, 12, 42)	Retrospective series (conservative)	1 g TID × 3 wks	—	Recurrence ∼5%–10%	—	—
TXA (40, 42)	RCT (Brazil), *n* = 50	—	No significant difference	—	—	Two thromboembolic events in TXA arm
Corticosteroids (DXM) (43–46)	DEX-CSDH RCT	16 mg/day taper × 14 days	Fewer re-operations	—	Worse 6-mo mRS; ↑ SAEs (hyperglycemia, infection, psychosis)	Significant adverse events
Corticosteroids (DXM) (12, 43–47)	Meta-analyses/observational	Variable	Mixed/neutral on recurrence	Some ↑ morbidity; signal toward ↑ mortality	—	Infections, GI bleed, hyperglycemia, VTE, psychosis reported

MGS, Markwalder Grading Scale; AEs, adverse events; RCT, randomized controlled trial; DXM, dexamethasone; VTE, venous thromboembolism.

Dose and duration: The best-studied regimen is atorvastatin 20 mg orally once nightly for 8 weeks, as used in the randomized, double-blind ATOCH trial; patients were then followed for 16 weeks off-drug. This dose was selected to balance efficacy with bleeding risk seen at very high statin doses in other cerebrovascular populations. In ATOCH, atorvastatin produced greater hematoma volume reduction at 8 weeks and fewer crossovers to surgery than placebo, without excess adverse events. In a separate phase-II randomized study exploring combination therapy, patients were assigned to atorvastatin 20 mg/day with or without a low-dose dexamethasone taper over 5 weeks (2.25 mg daily × 2 weeks → 0.75 mg BID × 2 weeks → 0.75 mg daily × 1 week); the combination arm showed larger radiographic improvement over 5 weeks, albeit in a small cohort. A subsequent protocolized trial (ATOCH-II) is testing 20 mg/day for 28 days with/without low-dose dexamethasone. Taken together, current evidence supports 20 mg/day for ∼8 weeks as a pragmatic starting regimen when conservative therapy is appropriate, while combination therapy remains investigational (34).

### 3.2 ATOCH phase II trial

Building on these encouraging data, a multicenter, double-blind, randomized controlled trial (ATOCH) randomized 200 patients with CT-confirmed cSDH and mild-to-moderate symptoms (Markwalder grades 1–2) to atorvastatin 20 mg daily vs. matched placebo for 8 weeks, followed by a 16-week off-treatment observation period (4).

The primary endpoint was the change in hematoma volume at 8 weeks; secondary endpoints included the Glasgow Outcome Scale–Extended, the rate of surgical intervention, and adverse events. Intention-to-treat analysis revealed a mean additional reduction of 12.5 mL in the atorvastatin arm compared to the placebo arm (95% CI: 0.9–23.9 mL; *p* = 0.003). Improvement in neurological function occurred in 45.9% of patients treated with atorvastatin vs. 28.6% of controls. (adjusted OR, 1.96, 95% CI: 1.07–3.58; *p* = 0.03). Moreover, the cumulative incidence of surgical rescue was significantly lower with atorvastatin (HR: 0.47, 95% CI: 0.24–0.92; *p* = 0.03). Drug tolerability was excellent, with no significant differences between groups in rates of myalgia, transaminase elevation, or other serious adverse events (35).

Subsequent Chinese investigations include the ATOCH II trial, which randomizes cSDH patients to atorvastatin plus low-dose dexamethasone vs. placebo to evaluate synergistic anti-inflammatory and vascular-stabilizing effects (36). Early laboratory data indicate that combined therapy is more effective in reducing membrane VEGF expression and improving endothelial barrier function compared with either agent alone (37). A small surgical pilot (*n* = 42) also reported faster postoperative hematoma clearance and lower recurrence rates when atorvastatin was administered perioperatively alongside steroids vs. steroids alone (38). A 2017 systematic review, conducted prior to the ATOCH RCT, concluded that statins show promise but underscored the need for randomized data (39).

## 4 Tranexamic acid use for cSDH treatment

Tranexamic acid (TXA) is a synthetic antifibrinolytic agent structurally similar to lysine. Its primary mechanism involves competitively inhibiting plasminogen activation and plasmin activity. It reversibly binds to lysine-binding sites on plasminogen, thereby preventing fibrin degradation (3, 4). In addition to its antifibrinolytic effects, TXA possesses anti-inflammatory properties, as plasmin activation can stimulate the kallikrein–kinin system, increasing vascular permeability (40).

Clinically, TXA has been shown to reduce bleeding across various contexts, including trauma, surgery, and uncontrolled hemorrhage (1). More recently, it has emerged as a potential adjunctive therapy for cSDH, aiming to stabilize the hematoma clot and prevent expansion, particularly following burr-hole evacuation. Several studies suggest that TXA not only reduces recurrence rates but also accelerates hematoma resolution by enhancing the resorption of residual clots (1, 2). A meta-analysis by Musmar et al. reported a significantly lower recurrence with TXA use (OR: 0.35, *p* < 0.01) and a marked reduction in hematoma volume at three months, without an associated increase in adverse events (OR: 1.84, *p* = 0.42) (3). Retrospective series have reported recurrence rates of 5%–10% in conservatively managed patients receiving oral TXA 1 g TID for three weeks (2). However, a Brazilian RCT (*n* = 50) found no significant difference in recurrence rates and reported two thromboembolic events in the TXA arm (41).

While preliminary data support TXA as both a postoperative adjunct and a conservative treatment option, current evidence remains insufficient, highlighting the need for large, high-quality trials to define its definitive role in optimizing cSDH outcomes. A European Phase III trial (*n* = 300) is currently underway to evaluate TXA in non-operatively managed cSDH, with stratification by baseline D-dimer and plasminogen activator levels. Results are anticipated in late 2025 (42) (Table 2).

## 5 Corticosteroid use for cSDH treatment

Corticosteroids have been investigated as a potential medical therapy for cSDH due to their ability to suppress the synthesis of proinflammatory cytokines, inhibit immune cell activity, downregulate proinflammatory enzymes, and reduce the production of nitric oxide and cyclooxygenase (4).These mechanisms are believed to confer anti-inflammatory, antifibrinolytic, and anti-angiogenic effects.

Glucocorticoids, primarily dexamethasone, have been evaluated for their ability to attenuate the inflammatory cycle within the cSDH membrane. Shrestha et al. reported 61% lower odds of cSDH recurrence in patients receiving dexamethasone compared with surgery alone (43). Small, nonrandomized studies suggested that perioperative dexamethasone reduces recurrence rates to 5%–8% when added to burr-hole drainage (44). However, other studies have yielded less favorable results. A meta-analysis by Almenawer et al. found no significant improvement in recurrence or cure rates with corticosteroid use, while noting that adjuvant therapy was associated with significantly higher morbidity (RR: 1.97, 95% CI: 1.54–2.45, *p* = 0.005) (45). Similarly, Wang et al. reported that dexamethasone increased the risk of all-cause mortality (RR 1.96, 95% CrI 1.20–3.28), with adverse events generally severe even at low doses. Kim and Lee also documented a higher mortality risk with dexamethasone compared to placebo (46).

Moreover, in the DEX-CSDH RCT, found that while dexamethasone (16 mg/day taper over 14 days) led to fewer reoperations, it worsened functional outcomes (mRS) at 6 months and increased serious adverse events (hyperglycemia 15%, infections 12%, steroid psychosis 3%) (47). These findings have led most centers to abandon routine steroid use except in select nonsurgical candidates or as a brief bridge in anticoagulated patients. Other reported adverse effects of corticosteroid therapy include infections (9% of patients), gastrointestinal bleeding, hyperglycemia (50% of diabetic patients and 3.8%–40% of non-diabetic patients), gastritis, pulmonary embolism, steroid-induced psychosis, cellulitis, pulmonary edema, and hypertension (48).

While steroids may help reduce recurrence in selected cases, their routine use is limited by the lack of proven benefit in functional outcomes and the potential for harm, emphasizing the need for tailored treatment decisions and further research.

## 6 Open surgeries (burr holes and Mini craniotomy)

### 6.1 Determinants of treatment approach

The choice of surgical technique for cSDH is influenced by multiple patient- and disease-specific factors. Key considerations include patient age, comorbidities, baseline functional status, and the use of anticoagulant or antiplatelet agents (49).

Hematoma characteristics such as size, midline shift, membrane organization, and density pattern on CT also guide decision-making. Neurological status, including the Glasgow Coma Scale (GCS) score and the presence of focal neurological deficits, is crucial for determining surgical timing and urgency (50). Finally, institutional expertise and surgeon preference can impact the selected approach (49, 50).

### 6.2 Overview of surgical management

The primary objective of surgical intervention is to evacuate the hematoma, alleviate the mass effect, and minimize recurrence in patients with focal neurological deficits or moderate to severe symptomatology. Common techniques include burr hole craniotomy (single or double burr holes) with or without closed-system subdural drainage, mini craniotomy with or without membranectomy or membrane fenestration, and, in selected cases, a wider craniotomy (51, 52).

Burr-hole evacuation is less invasive, associated with shorter operative times, and generally carries lower perioperative morbidity (53). It is the first-line approach for non-septated or low-complexity cSDHs and achieves low recurrence rates when combined with placement of a closed-system subdural drain for 24–48 h; a strategy that remains the gold standard for symptomatic cases (54, 55) (Table 3).

**Table 3:** Surgical and endovascular strategies.

Strategy	Typical indication	Drain use	Expected recurrence	Notable complications
Burr-hole(s) + closed-system drain (12, 46, 49–53, 58)	Symptomatic, non-septated CSDH	Yes (24–48 h)	∼10%–12%; RCT shows 9.3% with drain vs. 24% without	ICH (1%–3%), tension pneumocephalus (1%–2%), empyema (<1%)
Mini-craniotomy ± membranectomy (50, 52, 63, 64)	Organized/solidified, septated, or recurrent CSDH	Selective	May reduce recurrence in selected morphology	Longer OR time; potentially ↑ morbidity
Twist-drill craniostomy (49, 50, 52, 54, 55, 59)	Selected frail/bedside candidates	Variable	Higher recurrence if no drain; morphology-dependent	Suboptimal for septations
MMAE (adjunct or primary) (31, 56, 57, 66–70, 74)	High surgical risk, recurrence prevention, select primary therapy	N/A	Early trials suggest 50%–70% relative reduction in failure; e.g., 5% vs. 18% when adjunct to surgery	Stroke < 1%, cranial nerve palsy < 0.5%, scalp necrosis < 1%

In modern series employing routine drainage and standardized postoperative management, recurrence rates range from 10% to 12% (56, 57). A randomized controlled trial by Santarius et al. found that the use of drains after burr holes for cSDH had a significantly lower recurrence rate than without drains (9.3% vs. 24%, *p* = 0.003) (58). Furthermore, a case report by Kimura et al. found that failure to use a drain after burr hole evacuation of a subdural hygroma resulted in a cSDH that required another burr hole (13). Recurrence is often attributed to persistent outer membrane neovascularization and ongoing exudation rather than inadequate initial drainage.

Although serious complications such as intracerebral hemorrhage (1%–3%), tension pneumocephalus (1%–2%), and subdural empyema (<1%) are uncommon, frail elderly patients face markedly higher perioperative morbidity (up to 15% mortality in octogenarians) (59, 60). Furthermore, burr-hole drainage addresses mass effect but does nothing to modulate the inflammatory or angiogenic milieu that fosters hematoma persistence and growth.

The Subdural Evacuating Port System (SEPS) has emerged as a less invasive bedside alternative that can be performed under local anesthesia. SEPS involves a twist-drill craniostomy with the placement of a hollow port, allowing for continuous drainage of the hematoma into a closed system. This technique is particularly appealing for elderly or medically fragile patients in whom general anesthesia carries a high risk. While recurrence rates are somewhat higher compared to burr-hole drainage, SEPS offers lower perioperative complication rates and can often obviate the need for more invasive surgery in selected cases (61).

Mini craniotomy, by contrast, allows for more extensive removal of membranes and is preferred for organized, solidified, or septated hematomas, as well as for recurrent cases (53). While it may reduce recurrence in selected patients, it is associated with longer operative times and potentially higher morbidity (62). Comparative studies suggest similar functional outcomes when procedures are appropriately matched to the underlying hematoma morphology (63, 64).

Beyond the simple presence or absence of a drain, the type of drainage system may also influence outcomes. Subdural passive drains remain the most commonly used and were the standard in the landmark Santarius trial. However, alternative strategies such as active subgaleal drainage, where negative pressure is applied in a subgaleal location rather than within the subdural space, have recently gained attention. A large prospective series reported by Grubor et al. (2025) found that active subgaleal drains were associated with lower recurrence and infection rates compared with traditional subdural drains, potentially by reducing the risk of parenchymal injury and minimizing retrograde contamination. These findings suggest that drainage strategy should be considered not only in terms of duration but also system type, with active subgaleal drains representing a promising refinement of standard practice (65).

### 6.3 Practical implication

For symptomatic cSDH with mass effect, burr-hole evacuation with a closed subdural drain remains first-line because it reduces recurrence and mortality compared with no drain; many centers leave the drain in place for ∼24–48 h (55). Twist-drill craniostomy is a reasonable alternative in selected patients (e.g., significant comorbidity, bedside feasibility), with broadly comparable primary efficacy, though some studies report higher reoperation without suction drainage. Craniotomy with membranectomy is reserved for organized/loculated hematomas or repeated recurrence after less invasive approaches. Perioperative details matter: meticulous hemostasis, consideration of subdural irrigation and early mobilization are standard. Choice of surgical management and adjunct should be individualized by hematoma morphology (uni/bilateral, septations), neurological status, antithrombotic use, and anesthesia risk.

## 7 Middle meningeal artery embolization

**Figure 2:**
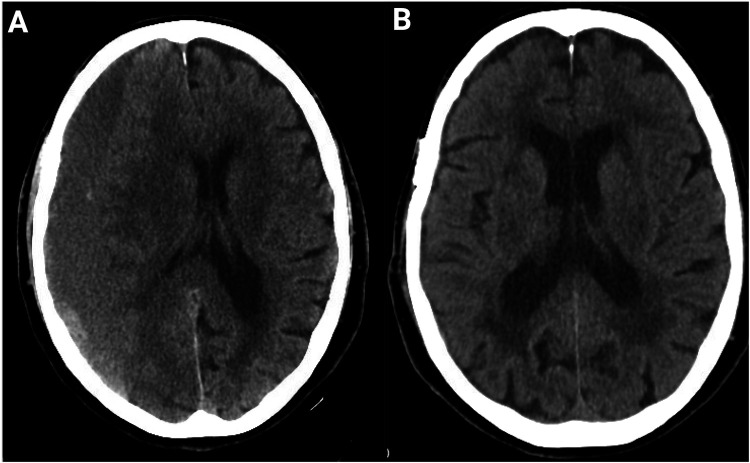
Resolution of CSDH post-adjuvant MMAE after burr hole treatment. **(A)** Axial CT head scan of an 89-year-old patient at presentation demonstrates a large chronic subdural hematoma with significant mass effect and midline shift. **(B)** Follow-up CT at 4 months after burr-hole drainage and adjunct MMAE using Onyx liquid embolic agent shows complete resolution of the hematoma with restoration of normal midline anatomy and no evidence of recurrence. No peri-procedural complications occurred.

Middle meningeal artery embolization (MMAE) has emerged as a promising minimally invasive adjunct or alternative to surgical evacuation (31, 66). MMAE targets the feeder vessels of the outer membrane, aiming to “starve” the neovasculature and halt further fluid exudation (67) (Figure 2). Indications include high surgical risk, prevention of recurrence after burr hole evacuation, preoperative embolization to reduce postoperative complications, and select cases as primary therapy (68).

MMAE is performed via transradial access, preferred over transfemoral access, to avoid complex femoral artery puncture using particulate or liquid embolic agents (68). Early evidence suggests that MMAE can substantially reduce recurrence rates compared to standard surgery (69). Preliminary randomized trials evaluating MMAE both as an adjunct to burr-hole drainage and as primary therapy for recurrent cSDH have reported relative reductions in treatment failure of 50%–70% (56, 70). Notably, Link et al. (2020) observed a recurrence rate of 5% with combined surgery + embolization vs. 18% with surgery alone (*p* = 0.01) in a cohort of 150 patients (70). While these findings are promising, ongoing randomized controlled trials are needed to define the procedure's precise role and optimal patient selection (69) (Table 3).

A variety of embolic agents have been utilized for MMAE, including particles (polyvinyl alcohol or microspheres), liquid embolics (Onyx, n-butyl cyanoacrylate), detachable coils, or combinations thereof (71). Particulate embolization is straightforward and widely available, but carries risk of distal migration and incomplete proximal occlusion. Liquid agents such as Onyx allow for deeper penetration of the MMA branches supplying the outer membrane, achieving durable occlusion, though they require meticulous technique to prevent reflux into collaterals. Coils are often used for proximal occlusion but may leave distal feeders patent if used alone, so they are frequently combined with particles or liquid embolics. Comparative studies have found no difference among liquid embolics in radiological improvement, need for surgical rescue, or major complications (71, 72).

Although early series suggest that combined approaches may reduce recurrence rates compared with single-agent strategies, current randomized trials are limited to liquid embolics, leaving the relative efficacy of combination techniques untested. Current practice patterns are heterogeneous, and ongoing randomized trials may help clarify the optimal embolic material for balancing efficacy with safety.

Complication rates in experienced centers are low, with ischemic stroke reported in <1%, cranial nerve palsy in <0.5%, and scalp necrosis in <1% of cases (56). One of the most feared adverse events is iatrogenic blindness, which occurs if embolic material inadvertently enters the ophthalmic circulation. This risk arises from dangerous anastomoses between the middle meningeal artery and branches of the ophthalmic artery, most commonly via the recurrent meningeal branch of the lacrimal artery or the anterior falcine artery. Embolization through these collaterals can occlude the central retinal artery, leading to irreversible vision loss (56). To minimize this risk, meticulous angiographic assessment for ophthalmic collaterals is mandatory prior to embolization, with microcatheter positioning performed distal to any potential anastomotic channels and careful, low-pressure injection of embolic material.

Another recognized though uncommon complication is facial nerve palsy, which may result from unintentional embolization of petrosal branches of the middle meningeal artery that contribute to the vascular supply of the facial nerve within the temporal bone (73). The risk can be mitigated by superselective catheterization of the target meningeal branches, avoidance of reflux, and use of embolic particles of appropriate size to reduce passage through small collaterals.

Recent randomized trials show that adjunctive MMAE lowers recurrence/treatment-failure when added to standard care (both after surgical evacuation and, in selected non-acute cases, as part of initial management) without a short-term increase in disabling stroke or death. That said, MMAE is not for every patient. It is most compelling when recurrence risk is high (e.g., anticoagulation/antiplatelet use, bilateral disease, significant membrane vascularity, prior recurrence) or surgical/anesthesia risk is unfavorable. By contrast, patients with acute hyperdense components, marked mass effect with deterioration, or inaccessible/unsafe vascular anatomy are better served by prompt surgical evacuation; MMAE can be considered later as an adjunct to reduce recurrence (74). Guidance is evolving, NICE (UK) currently restricts MMAE to research settings pending more data, while expert consensus and multiple RCTs support it as a potent adjunct in neurologically stable, non-acute cSDH to reduce recurrence. In short, MMAE should be offered selectively, not universally, with decisions anchored to trial-style inclusion criteria, local expertise, and patient preferences.

## 8 Emerging therapies & future directions

Despite advances in understanding cSDH biology, existing therapies are largely reactive, focused on evacuating accumulated blood rather than targeting the underlying membranes that drive recurrence. Recurrence rates of 10%–20% across modalities, coupled with risks inherent to both surgery and pharmacotherapy, underscore the need for disease-modifying strategies. A truly disease-modifying approach to cSDH will require therapies that go beyond simple evacuation of the fluid collection and instead target the underlying membrane biology.

One promising avenue is biomarker-guided personalization of treatment. Recent analyses of cSDH fluid and paired serum samples have identified elevated levels of angiogenic and inflammatory mediators, particularly VEGF, IL-6, and IL-8, as well as markers of fibrinolysis, such as D-dimer and plasminogen activator inhibitor-1 (PAI-1) (75). By quantifying these biomarkers at presentation, clinicians could stratify patients into high-risk vs. low-risk recurrence groups: for example, a patient whose hematoma fluid demonstrates markedly elevated VEGF and IL-6 might be flagged for early adjunctive anti-angiogenic therapy (such as a statin or an anti-VEGF monoclonal antibody), whereas a patient with low inflammatory and fibrinolytic activity might safely undergo standard burr-hole drainage without additional pharmacotherapy. Prospective validation of such a “cSDH inflammation index” is currently underway at several centers, with the goal of developing a point-of-care assay that can guide real-time treatment decisions.

Building on the central role of VEGF in outer membrane neovascularization, anti-VEGF agents have been explored in small, compassionate-use settings. A case series of five patients with refractory, recurrent cSDH treated off-label with intravenous bevacizumab reported an average hematoma volume reduction of over 80% at 6 weeks, without the need for further surgery (76). Patients tolerated bevacizumab well, with only mild hypertension in two cases and no proteinuria or thromboembolic events. Nevertheless, bevacizumab carries risks of hypertension, impaired wound healing, and rare gastrointestinal perforation, so formal phase II trials are needed to define optimal dosing, duration, and patient selection criteria before anti-VEGF therapy can be recommended outside of experimental protocols.

An alternative strategy is local immunomodulation, which aims to concentrate anti-inflammatory and reparative factors directly at the site of disease, thereby sparing the rest of the body from systemic side effects. Two approaches are under active investigation. The first is the intrathecal or subdural administration of corticosteroids: small animal studies suggest that delivering a low dose of dexamethasone into the subdural space can attenuate membrane cytokine expression, with negligible increases in blood glucose levels or risk of infection. The second approach involves injecting platelet-rich plasma (PRP) into the subdural space during burr-hole drainage. PRP is rich in growth factors (PDGF, TGF-β) that promote endothelial stabilization and fibroblast proliferation.

On the surgical front, neuroendoscopic membranectomy is gaining traction to remove the pathological outer membrane directly. Whereas traditional burr-hole drainage evacuates fluid but leaves much of the membrane intact, endoscopic techniques allow for the visualization of septations and membrane folds, facilitating a more complete removal. Early case series (*n* = 30) report recurrence rates of less than 5% at one year, compared with 12% in matched historical cohorts (77). However, widespread adoption is limited by the need for specialized endoscopic equipment and training, as well as longer operative times.

## 9 Conclusion

cSDH is a membrane-mediated disorder in which persistent inflammation, aberrant angiogenesis, and local fibrinolysis drive hematoma expansion and recurrence. While surgical intervention remains the cornerstone of treatment, its limitations underscore the need for disease-modifying strategies. Pharmacologic agents, such as statins, tranexamic acid, and corticosteroids, as well as minimally invasive approaches like middle meningeal artery embolization, show promise; however, current evidence remains limited. Further elucidation of the subdural membrane pathophysiology, along with well-designed randomized controlled trials, is essential to establish targeted pharmacological and endovascular therapies as viable, patient-specific treatment options.
